# North American Public Opinion Survey on the Acceptability of Crowdsourcing Basic Life Support for Out-of-Hospital Cardiac Arrest With the PulsePoint Mobile Phone App

**DOI:** 10.2196/mhealth.6926

**Published:** 2017-05-17

**Authors:** Katie N Dainty, Haris Vaid, Steven C Brooks

**Affiliations:** ^1^ Rescu Li Ka Shing Knowledge Institute St Michael's Hospital Toronto, ON Canada; ^2^ Institute of Health Policy Management and Evaluation University of Toronto Toronto, ON Canada; ^3^ School of Medicine Queen's University Kingston, ON Canada; ^4^ Department of Emergency Medicine Queen's University Kingston, ON Canada

**Keywords:** sudden cardiac death, surveys and questionnaires, cardiopulmonary resuscitation, PulsePoint, North America

## Abstract

**Background:**

The PulsePoint Respond app is a novel system that can be implemented in emergency dispatch centers to crowdsource basic life support (BLS) for patients with cardiac arrest and facilitate bystander cardiopulmonary resuscitation (CPR) and automated external defibrillator use while first responders are en route.

**Objective:**

The aim of this study was to conduct a North American survey to evaluate the public perception of the above-mentioned strategy, including acceptability and willingness to respond to alerts.

**Methods:**

We designed a Web-based survey administered by IPSOS Reid, an established external polling vendor. Sampling was designed to ensure broad representation using recent census statistics.

**Results:**

A total of 2415 survey responses were analyzed (1106 from Canada and 1309 from the United States). It was found that 98.37% (1088/1106) of Canadians and 96% (1259/1309) of Americans had no objections to PulsePoint being implemented in their community; 84.27% (932/1106) of Canadians and 55.61% (728/1309) of Americans said they would download the app to become a potential responder to cardiac arrest, respectively. Among Canadians, those who said they were likely to download PulsePoint were also more likely to have ever had CPR training (OR 1.7, 95% CI 1.2-2.4; *P*=.002); however, this was not true of American respondents (OR 1.0, 95% CI 0.79-1.3; *P*=.88). When asked to imagine themselves as a cardiac arrest victim, 95.39% (1055/1106) of Canadians and 92.44% (1210/1309) of Americans had no objections to receiving crowdsourced help in a public setting; 88.79% (982/1106) of Canadians and 84.87% (1111/1309) of Americans also had no objections to receiving help in a private setting, respectively. The most common concern identified with respect to PulsePoint implementation was a responder’s lack of ability, training, or access to proper equipment in a public setting.

**Conclusions:**

The North American public finds the concept of crowdsourcing BLS for out-of-hospital cardiac arrest to be acceptable. It demonstrates willingness to respond to PulsePoint CPR notifications and to accept help from others alerted by the app if they themselves suffered a cardiac arrest.

## Introduction

### Background

Each year, more than 400,000 people have out-of-hospital cardiac arrest (OHCA) in the United States and Canada. Early cardiopulmonary resuscitation (CPR) and defibrillation are key links in the chain of survival for OHCA. Studies in specific high-risk community settings, such as casinos [1], airports [2], and aboard aircraft [3], have reported increased survival with the implementation of CPR and automated external defibrillator (AED) training, along with an organized response to cardiac arrest emergencies. Evidence from the “public access defibrillation (PAD) trial,” which randomized 993 community units, such as apartment complexes and community centers, demonstrated that an organized lay-responder PAD program, including CPR training, can double the chance of survival for individuals who suffer OHCA [4]. Many historical approaches to increasing bystander resuscitation are limited by the fact that bystanders are chosen by circumstance, and not design. Many OHCA victims have probably died without receiving the benefit of bystander resuscitation, while trained, capable, and willing rescuers were nearby, but just out of sight.

### Crowdsourcing Basic Life Support for OHCA

Crowdsourcing has been defined as the process of “obtaining needed services, ideas, or content by soliciting contributions from a large group of people, and especially from the online community, rather than from traditional employees or suppliers” [5]. The PulsePoint Respond mobile device application (app) is an example of a technology solution applying the concept of crowdsourcing to the problem of OHCA. The PulsePoint Respond mobile device app uses crowdsourcing to address the problem of OHCA. In communities where PulsePoint has been implemented, citizens can download PulsePoint Respond onto their mobile device from the Apple App Store or Google Play free of charge. Minimal contact information is collected on each user to ensure privacy and confidentiality. A major component of PulsePoint implementation involves a coordinated communications and public outreach strategy, encouraging CPR-trained individuals to download the PulsePoint Respond app onto their mobile devices. Messaging, which specifically targets individuals with CPR training (eg, paramedics, emergency medical technicians, firefighters, other health care providers, and CPR course graduates), is supported by resources from the PulsePoint Foundation and is generally coordinated by a communications professional within the hosting public safety agency. By using the global positioning system, mapping functionality of mobile devices, along with cardiac arrest location data provided by local 911 emergency call centers, the PulsePoint system can send directed cardiac arrest notifications to PulsePoint Respond users in close proximity (default 400 m) to the event. The notifications include the exact location of suspected cardiac arrest emergencies and registered AEDs [6].

### Study Objective

PulsePoint engages community volunteers outside of the traditional professional emergency response as users. Although the majority of PulsePoint volunteers are off-duty health care providers [7], anyone in the community can download the app and become a PulsePoint responder. When new users download the app, they are asked to declare that they are CPR trained and willing to respond in an emergency. Designed as a true crowdsourcing solution to remove barriers to registration and encourage large numbers of “good samaritans” to participate, this self-declaration is not vetted. This design is consistent with the general understanding that any attempt as resuscitation during cardiac arrest, whatever the quality, is better than no attempt at all. The traditional response to 911 calls for emergency medical conditions has been limited to professional emergency personnel with validated CPR training. PulsePoint represents a departure from the status quo and has raised concerns about privacy and public safety among public safety agencies approached to consider implementation.

These are potential barriers to implementation of crowdsourcing solutions for OHCA. It is not clear whether the public perception of crowdsourcing for emergency response to cardiac arrest, specifically around privacy and safety issues, is consistent with the privacy and liability concerns that may be harbored by decision makers in public safety agencies. This valuable information could guide future research and implementation efforts. Accordingly, our study objective was to evaluate the public perceptions of the PulsePoint mobile app in the North American setting. More specifically, the aim of this research was to determine the level of public acceptance of crowdsourcing basic life support (BLS) for cardiac arrest and identify specific concerns with this strategy.

## Methods

A Web-based public opinion survey was conducted within Canada and the United States in collaboration with an established polling vendor, IPSOS Reid. This study was approved by the Queen’s University Health Sciences Research Ethics Board.

### Setting

This study used the IPSOS eNation Canadian and US Online Omnibus survey platforms [8]. Data were collected through stratified random sampling of the more than 800,000-member Web-based panel and the Ampario sample source. The IPSOS Web-based panel is recruited and maintained using double and triple opt-in screening processes to ensure maximum return from an engaged and representative audience. The panel is updated regularly and non-responders are removed.

### Study Sample

The study sample was designed to be nationally representative of the adult Canadian and American populations. Data were weighted on gender, age, region, and income, based on census information, to ensure that the sample’s composition reflected that of the reference population. The Canadian sampling process included an additional sampling of French-speaking respondents in Canada to provide a base for analysis within that group. The US sampling process included an additional sample of Spanish-speaking respondents to provide a base for analysis within that group. The margin of error associated with this technique on a sample size of 1000 adults is <±3.1% relative to the result that would be attained after polling the entire population, 19 times out of 20 [9].

### Study Questionnaire

We presented the respondents with a short concept description of cardiac arrest and the PulsePoint app, followed by six closed-ended and four open-ended questions (Multimedia Appendix 1). Survey questions were developed by the research team and experts from IPSOS-Reid and pilot tested with a group of six lay public members affiliated with our research program. Our primary outcome was comfort level with the idea of having a PulsePoint-notified responder attend the respondent, imagining the scenario that they had suffered a sudden cardiac arrest. We specifically asked them about their comfort level when considering the situation of cardiac arrest in a public location versus a private (residential) location.

### Data Analysis

**Figure 1:**
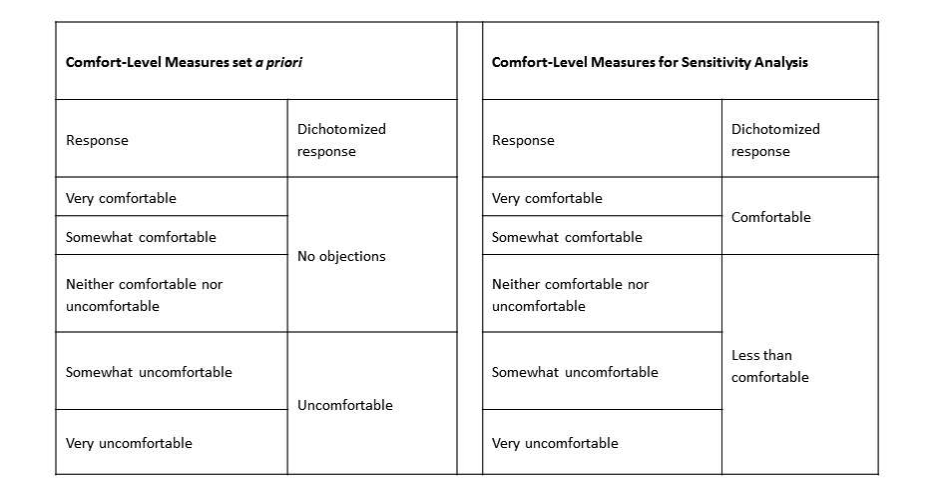
Dichotomization of comfort level measures as reported by survey respondents for logistic regression analysis and sensitivity analysis.

Survey responses from the United States and Canada were treated independently because the samples were individually weighted according to US or Canadian census data. Analysis of the survey responses was carried out using descriptive statistics and comparison of groups based on responses to primary questions using chi-squared testing. We considered a *P* value of less than .05 to be statistically significant. We also conducted weighted-logistic regression analysis to assess the relationship between demographic factors (ie, the predictor variables) and two outcome variables (STATA version 13, STATA Corp). The weights used in our regression analysis were the same weights used to design the study sample, as described earlier. The outcome variables were the respondent’s comfort level to crowdsourced BLS for an OHCA in a public location or a private location (see Questions 4 and 6 in Multimedia Appendix 1). Responses to the outcome variables, which were reported on a Likert scale, were dichotomized with specified cut-points set *a priori*. This was done to simplify the interpretation of the weighted-logistic regression analysis. Individuals who responded to the comfort-level questions with “very comfortable,” “somewhat comfortable,” or “neither comfortable nor uncomfortable” were coded as having “no objections.” Individuals who responded with “somewhat uncomfortable” and “very uncomfortable” were coded as feeling “uncomfortable” (Figure 1).

A planned sensitivity analysis was conducted by using a different cut-point in the dichotomization of the outcome variables. Individuals who responded to the comfort-level questions with “very comfortable” and “somewhat comfortable” were coded as feeling “comfortable,” while individuals who responded with “neither comfortable nor uncomfortable,” “somewhat uncomfortable,” and “very uncomfortable” were coded as feeling “less than comfortable” (Figure 1). The sensitivity analysis was conducted to test the stability of our primary regression models. We wanted to make sure that dichotomizing at a given cut-point, versus another cut-point, would not significantly influence the interpretation of our analysis. We built separate univariable and multivariable weighted logistic regression models for each outcome (comfort in private settings, comfort in public settings) in each national cohort (United States and Canada). Multivariable regression models included the following predictor variables: sex, education, household income, employment status, whether or not the respondent had children, age group, spoken language (Canadian data only), marital status (US data only), and race (US data only). We did not apply the finite population correction because our sample size was significantly smaller than the inference population. Open-ended, text-based responses regarding concerns were coded and summarized using standard content analysis [10].

## Results

Data were collected between May 29 and June 3, 2015. A total sample of 2415 total surveys was collected: 1106 from Canada and 1309 from the United States. We did not calculate the response rate as the nature of the panel survey approach renders this statistic inapplicable. The demographic characteristics of the respondents are outlined in Table 1. A summary of the survey responses is presented in Table 2. At some point, 70% (769/1106) of Canadians and 63% (828/1309) of Americans had been trained in CPR. However, about one-third obtained their certification more than five years ago (United States: 441/1309, 34%; Canada: 356/1106, 32%). Among Canadians, those who said that they were likely to download PulsePoint were also more likely to have ever had CPR training (OR 1.7, 95% CI 1.2-2.4; *P*=.002). This suggested that knowledge of CPR might influence the likelihood of registering as a PulsePoint user. However, this relationship did not hold true among Americans (OR 1.0, 95% CI 0.79-1.3; *P*=.88).

Most Canadians (1088/1106, 98%) and Americans (1259/1309, 96%) had no objections to PulsePoint implementation in their community. Furthermore, most had no objections to receiving CPR if they were victim of cardiac arrest in a public location (Canada: 1055/1106, 95%; United States: 1210/1309, 92%) or private location (Canada: 982/1106, 89%; United States: 1111/1309, 85%).

Among Americans and Canadians, after adjusting for covariates, multivariable regression analyses found that demographic factors were not associated with comfort-level measures in the *public setting* scenario (Multimedia Appendices 2 and 3). Sensitivity analysis, with different cut-off points for the dichotomous outcome variables used in each model, revealed similar results. The multivariable regression analysis assessing comfort level to crowdsourced BLS in a *private setting* found that, compared with females, males had higher odds of having no objections to crowdsourced BLS (United States: OR 1.7, 95% CI 1.2-2.4; *P*=.004; Canada: OR 2.3, 95% CI 1.3-3.9) (Multimedia Appendices 2 and 3). Among Americans, compared with single individuals, married or co-habitating individuals had 1.7 times (95% CI 1.1-2.8; *P*.03) the odds of having no objections to crowdsourced BLS in a *private setting* scenario (Multimedia Appendix 2). Among Canadians, in comparison to individuals with household income <$50,000, those with income between $50,000 and $99,999 had half the odds of having no objections to crowdsourced BLS in a *private setting* scenario (OR 0.51, 95% CI 0.28-0.92, *P*=.02; Multimedia Appendix 3). However, this finding was not robust to the outcome cut-off selection; the odds ratio for this covariate was not statistically significant in the sensitivity analysis.

**Figure 2:**
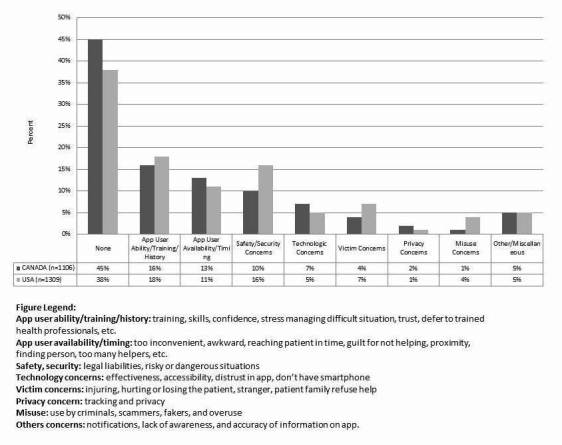
Concerns about the implementation of the Pulsepoint™ application by category.

When asked, less than 60% (1421/2415) of the respondents identified any concerns with the PulsePoint mobile device app as described. The results are summarized in Figure 2. The most common concerns raised by the respondents were lack of training among PulsePoint responders and trust issues (Canada: 174/1106, 16%; United States: 241/1309, 18%). This was largely driven by concerns that PulsePoint responders might not have sufficient ability or training to provide effective assistance (Canada: 140/1106, 13%; United States: 190/1309, 15%). Some expressed security concerns, including legal liabilities, especially among Americans, and being stuck in a risky or dangerous situation. When asked to consider cardiac arrest in a public setting, concerns around lack of training and ability were the most significant here as well (Canada: 235/1106, 21%; United States: 350/1309, 27%). Security (Canada: 111/1106, 10%; United States: 215/1309, 16%) and privacy (Canada: 28/1106, 2%; United States: 9/1309, 1%) issues were less significant concerns. When asked to consider cardiac arrest in a private (ie, residential) setting, lack of training and ability remained the most significant concern (Canada: 165/1106, 15%; United States: 244/1309, 19%). Security (Canada: 131/1106, 12%; United States: 173/1309, 13%) and privacy issues (Canada: 129/1106, 12%; United States: 159/1309, 12%) were also generally more significant concerns in this setting.

Among Canadians and Americans, 90% (995/1106) and 89% (1158/1309) felt that it was important that responders have up-to-date CPR certification, respectively. This is even more pronounced among those who say they would download the app (Canada: OR 5.2, 95% CI 3.0-9.1; *P*<.001; United States: OR 4.3, 95% CI 2.8-6.6; *P*<.001).

**Table 1:** Respondent demographics.

Demographics		Canada (N=1106), n (%)	United States (N=1309), n (%)	*P* value
**Gender**				
	Male	540 (49)	579 (44)	.05
	Female	566 (51)	730 (56)	
**Age (in years)**				
	18-34	315 (28)	381 (29)	.94
	35-54	416 (38)	484 (37)	
	55+	375 (34)	443 (34)	
**Primary language**				
	English	838 (76)	1153 (88)	-
	Spanish^a^	-^c^	156 (12)	
	French	268 (24)	-	
**Education**				
	Less than high school	71 (6)	49 (4)	<.001
	High school diploma	210 (19)	277 (21)	
	Post-secondary	466 (42)	475 (36)	
	University degree	359 (32)	509 (39)	
**Household income**				
	< $50,000	304 (27)	438 (33)	<.001
	$50,000-99,000	335 (30)	602 (46)	
	$100,000-149,000	252 (23)	179 (14)	
	$150,000 +	72 (7)	90 (7)	
**Marital status^b^**				
	Single	-	314 (24)	-
	Married or cohabitating	-	799 (61)	
	Widowed	-	61 (5)	
	Divorced or separated	-	135 (10)	
**Children**				
	Yes	315 (28)	347 (26)	.34
	No	791 (72)	962 (74)	
**Completed CPR training**				
	Ever	769 (70)	828 (63)	.003
	Within the last year	128 (12)	154 (12)	<.001
	In the past 5 years	286 (26)	233 (18)	
	> 5 years ago	356 (32)	441 (34)	

^a^Data on the proportion of Spanish-speaking Canadians were not available for Canadian respondents.

^b^Data on marital status were not available for Canadian respondents.

^c^Hyphens indicate that the data were not collected.

**Table 2:** Survey responses by question.

Question		Canada, n (%)	United States, n (%)	*P* value
**To what extent do you agree or disagree that the PulsePoint Respond app is something that you would want to be made available in your community?**				
		N=1106	N=1309	
	Agree or strongly agree	903 (82)	1005 (77)	.002
	Neither agree nor disagree	186 (17)	254 (19)	
	Disagree or strongly disagree	18 (1)	50 (4)	
**If the PulsePoint app was available in your community, how likely are you to download the app onto your mobile device?**				
		N=932	N=1189	
	Likely or very likely	583 (62)	728 (61)	.26
	Neither likely nor unlikely	183 (20)	214 (18)	
	Unlikely or very unlikely	166 (18)	247 (21)	
**If you suffered a cardiac arrest in a *public setting* (eg, walking down the street, in a park, at the mall, at work), how comfortable would you be with nearby PulsePoint users being notified of your exact location and coming to help you until professional crews arrived?**				
		N=1106	N=1309	
	Comfortable or very comfortable	882 (80)	1002 (77)	.02
	Neither comfortable nor uncomfortable	174 (16)	207 (16)	
	Uncomfortable or very uncomfortable	51 (5)	99 (8)	
**If you suffered a cardiac arrest in a *private setting* (eg, your home or the home of a friend or relative), how comfortable would you be with nearby PulsePoint users being notified of your exact location and coming to help you until professional crews arrived?**				
		N=1106	N=1309	
	Comfortable or very comfortable	796 (72)	892 (68)	.04
	Neither comfortable nor uncomfortable	187 (17)	219 (17)	
	Uncomfortable or very uncomfortable	124 (11)	198 (15)	
**How important is it to you that all PulsePoint users, who could potentially be notified of cardiac arrest locations, have a valid and up-to-date CPR certification?**				
		N=1106	N=1309	
	Important or very important	995 (90)	1158 (89)	.54
	Neither important nor unimportant	93 (8)	124 (9)	
	Unimportant or very unimportant	18 (2)	27 (2)	

## Discussion

### Principal Findings

In a representative sample of the North American public, we found that most are in favor of crowdsourcing BLS for cardiac arrest in their community with the PulsePoint system. In addition, we found that most have no objections with the concept of receiving help from anonymous PulsePoint users in the setting of a public location cardiac arrest emergency, occurring outside of the hospital. When prompted, a minority of respondents raised concerns regarding issues around training and capability of PulsePoint responders, safety, and privacy. These results provide important insight for future implementation of such systems.

Crowdsourcing is increasingly being used to address challenges in health care research and delivery. Professional researchers crowdsource cohorts from health social networks for conducting traditional studies regularly so they can quickly and efficiently get opinions and test potential interventions [11-14]. For example, a new Web-based program, CrowdMed, aims to leverage the “wisdom of the crowd” by giving patients an opportunity to submit their cases of undiagnosed illnesses and interact with case solvers to obtain diagnostic possibilities [14]. Scientists from the University of Southampton and The University of Pennsylvania Perelman School of Medicine ran a “My Heart Map Challenge” to create a map of automatic external defibrillators (AEDs) in the city of Pittsburgh. The map was populated by members of the general public identifying and submitting photos and information about public AEDs [15].

Traditionally, 911 calls result in a response from vetted on-duty professional responders. PulsePoint and other similar systems represent a paradigm shift in the way in which we deploy help for 911 callers reporting a possible cardiac arrest and provide the ability to connect responders and victims in a timelier way to address the issue of low bystander response rates. Recently, Ringh et al found that rates of bystander-initiated CPR could be significantly increased with the use of a similar mobile phone positioning system. That system also located mobile phone users and dispatched lay volunteers who were trained in CPR to a patient nearby with OHCA [16]. Zijlstra et al and Pijls et al had similar findings in their studies of SMS text message (short message service, SMS) alert systems in Sweden and the Netherlands, respectively [17,18]. However, there are challenges with such systems, including low response rates, misidentification of cardiac arrest victims, and technical difficulties, such as excessive activation radii, and insufficient user density in the community [18]. As such, these systems have not received much scientific attention. During recent attempts to implement the PulsePoint system in several Canadian jurisdictions because of concerns over protecting privacy and uncertainty about public opinion or potential backlash [7]. Our work demonstrates that public opinion strongly supports the implementation of PulsePoint-type apps and that many members of the public are willing to become responders, if given the opportunity.

Technologies are not separate from the society in which they are embedded, but are rather integral to the advancement of the social environment [19]. Society’s increasing dependence on technologies, however, comes with an increased need to closely examine “society-technology” interactions. While on the one hand, a new technology may bring about radical changes in society, on the other hand, the fate of that technology rests with the society in which it is being applied. Much research has been conducted on risk and benefit perceptions and public attitudes, as these are believed to be the major factors influencing public acceptance of technologies [20-24]. In a review by Gupta et al on the sociopsychological determinants of public acceptance of technologies, perceived risk, perceived benefit, trust and culpability, knowledge, individual differences, and attitude are traditionally the most often reported or cited determinants [19]. This maps closely with the domains we included in our study. The most common concern identified with respect to PulsePoint implementation was a potential crowdsourced responders’ lack of ability, training, knowledge, or having proper equipment in a public setting. In the case of PulsePoint, our results would indicate that the general public feels that the perceived benefit outweighs any perceived risk. Further, while issues of trust and privacy exist, in general, people would find it more than acceptable to use and to receive help from other users.

Findings from this work have several implications for the design of new crowdsourcing apps for cardiac arrest response or improvement of existing apps of this nature. For example, to address the concerns raised by respondents regarding the knowledge of responders, designers may consider including a training requirement to download the application. This could be as informal as agreeing to watch a short 5-minute video before download or as formal as uploading proof of professional CPR training. Each may have an impact on the public’s willingness to download and would need to be monitored. Apps may also need to consider collecting further registration information from responders in order to ensure the safety of victims as well as to gather information on bystander response, something that is currently very difficult to collect.

### Limitations

As with any survey-based study, our research has limitations. Our survey was conducted using a Web-based survey platform, which may create a bias toward individuals with computer literacy and access to the Internet. Due to the nature of the panel survey approach, we were unable to calculate the traditional response rate. Opinions may differ between responders and nonresponders to the survey, so our results may have been subject to selection bias. Finally, our weighted-logistic regression analysis was limited by a small sample size (within variables). This may limit the precision and power of our measures of association. Thus, although an association may not have been found in these analyses, a relationship between certain demographic characteristic and comfort level to crowdsourced BLS in a public or private setting may still exist due to type-two error.

### Conclusions

A key conclusion of the American Heart Association statement on the use of mobile devices, social media, and crowdsourcing as digital strategies to improve emergency cardiovascular care is that there is a clear need for rigorous research to build the scientific evidence base for their effectiveness and safety [25]. Our findings provide the first empirical scientific evidence that the North American public supports the implementation of the PulsePoint mobile device application to crowdsource BLS for OHCA. The large majority of people are comfortable with the concept of receiving BLS from nearby PulsePoint users when given the hypothetical situations of suffering a cardiac arrest in a public or private location. Concerns around PulsePoint responders’ abilities, level of training, and personal safety should be considered when planning implementation in communities as well as when designing future versions of the PulsePoint system. The results of our analysis should be very useful for decision-makers considering implementation of this strategy. Our findings can also guide developers of crowdsourcing solutions in addressing concerns related to trust and responder training in order to optimize community uptake.

## Appendix

Multimedia Appendix 1 Survey questionnaire.

Multimedia Appendix 2 Weighted regression analysis for the USA population.

Multimedia Appendix 3 Weighted regression analysis for the Canadian population.

## Abbreviations

AED: automated external defibrillator
AEDs: automatic external defibrillators
BLS: basic life support
CPR: cardiopulmonary resuscitation
OHCA: out-of-hospital cardiac arrest
PAD: public access defibrillation
