# Supplementary material for: North American Public Opinion Survey on the Acceptability of Crowdsourcing Basic Life Support for Out-of-Hospital Cardiac Arrest With the PulsePoint Mobile Phone App
Source: JMIR Mhealth Uhealth. 2017 May 17;5(5):e63. doi: 10.2196/mhealth.6926 (PMC5451638; doi:10.2196/mhealth.6926)
Supplement: Multimedia Appendix 1 [file mhealth_v5i5e63_app1.pdf]

SAMPLE SPECS

- The study will be collected as part of the Ipsos Online Omnibus
- The study will be conducted with nationally representative samples of adult Canadians and Americans
- A total of n=1,000 online interviews will be conducted per country with boosts of n=100 French-speaking Canadians within Canada and n=230 Spanish-speaking Americans within the US (in an effort to achieve approximately n=300 completes among each of these two groups).

SECTION 1: Screening & Intro

Q1. How recently, if ever, have you completed CPR training? (Please select one)
[DOWN]
Within the last year
2 to 3 years ago
3 to 4 years ago
4 to 5 years ago
More than 5 years ago
I have never received CPR training

[INFO SCREEN] Please read the following description carefully in order to answer the questions that follow.

A "Sudden cardiac arrest" occurs when someone's heart stops beating unexpectedly. Unless the heart can be restarted quickly, people who have suffered a cardiac arrest will die. "Cardiopulmonary resuscitation" or "CPR" involves pushing hard and fast on a victim's chest to mimic the action of the heart.

When 911 is called for this situation, paramedics and firefighters rush to the scene. However, emergency crews usually take more than 5 minutes to arrive after a 911 call. This is too long to wait. **Chances of survival decrease by 10% for each minute that CPR is not provided**. This is why bystanders who see event are so important. Doing CPR while professional rescuers are en route can quadruple the odds of survival. The problem is that currently, bystanders provide CPR in only 3 of every 10 cardiac arrest cases. Many people suffer cardiac arrest in our community and do not receive the benefit of bystander CPR because trained individuals are unaware that an arrest has occurred nearby.

The **Pulsepoint Respond** smartphone application aims to improve this situation. Anyone can sign up to be a PulsePoint responder by downloading the free app to your mobile device. Application users who indicate they are trained in (CPR) and would be willing to provide CPR in the event of an emergency are notified by the app when the device is within 400 meters of a suspected cardiac arrest reported to 911. The cardiac arrest notifications cause the device to ring and also provide the user with a detailed map showing the location of the emergency and any nearby public access defibrillators. The notification includes the exact address of the emergency but does not contain any specific patient information such as name, gender or age. The PulsePoint Respond cardiac arrest alerts are sent simultaneously with the dispatch of professional responders such as ambulance and fire crews.

Q2. Based on this description, to what extent do you agree or disagree that the PulsePoint Respond application is something that you would want to be made available in your community?
[DOWN]
Strongly agree
Somewhat agree
Neither agree nor disagree

Somewhat disagree
Strongly disagree

Q3. [ASK IF Q2 = somewhat disagree or completely disagree] And why is it that you would not want the PulsePoint Respond application to be made available in your community?
[CAPTURE OPEN END RESPONSE]

ASK SECTIONS 2 AND 3 IN RANDOM ORDER.

SECTION 2: Cardiac Arrest Victim

[RANDOMIZE THE ORDER OF SECTIONS 2.1 AND 2.2]

[TEXT TO PRECEDE FIRST OF TWO SECTION] Thinking of yourself as a **Cardiac Arrest Victim** and potential recipient of help/CPR from someone alerted by the PulsePoint app, please answer the following questions:

[TEXT TO PRECEDE SECOND OF TWO SECTION] Continuing to think of yourself as a **Cardiac Arrest Victim** and potential recipient of help/CPR from someone alerted by the PulsePoint app, please answer the following questions:

SECTION 2.1: Public Setting

Q4. If you suffered a cardiac arrest in a **Public Setting** (e.g. walking down the street, in a park, at the mall, at work, etc.), how comfortable would you be with nearby PulsePoint users being notified of your exact location and coming to help you until professional crews are arrived?
[ACROSS]
Very comfortable
Somewhat comfortable
Neither comfortable nor uncomfortable
Somewhat uncomfortable
Very uncomfortable

Q5. If you suffered a cardiac arrest in a **Public Setting** what concerns, if any, would you have if nearby PulsePoint users were notified of your exact location and came to help you until professional crews arrived? (Please be as detailed as possible.)
[CAPTURE OPEN END RESPONSE]

SECTION 2.2: Private Setting

Q6. If you suffered a cardiac arrest in a **Private Setting** (e.g. your home or the home of a friend or relative), how comfortable would you be with nearby PulsePoint users being notified of your exact location and coming to help you until professional crews arrived?
[ACROSS]
Very comfortable
Somewhat comfortable
Neither comfortable nor uncomfortable
Somewhat uncomfortable
Very uncomfortable

Q7. If you suffered a cardiac arrest in a **Private Setting** what concerns, if any, would you have if nearby PulsePoint users were notified of your exact location and came to help you until professional crews arrived? (Please be as detailed as possible)
[CAPTURE OPEN END RESPONSE]

SECTION 2.3: Final question for cardiac victims

Q8. How important is it to you that all PulsePoint users, who could potentially be notified of cardiac arrest locations, have a valid and up-to-date CPR certification?
[ACROSS]
Very important
Somewhat important
Neither important nor unimportant
Somewhat unimportant
Very unimportant

SECTION 3: Potential User of PulsePoint

[INFO SCREEN] Thinking of yourself as a **potential user of the Pulsepoint App** please answer the following questions.

Q9. If the PulsePoint app was available in your community, how likely are you to download the app onto your mobile device?
[ACROSS]
Very likely
Somewhat likely
Neither likely nor unlikely
Somewhat unlikely
Very unlikely
I do not have a mobile device

Q10. Do you have any particular concerns when thinking about being a PulsePoint Respond user notified of a nearby cardiac arrest? (Please be as detailed as possible.)
[CAPTURE OPEN END RESPONSE]
