# Supplementary material for: North American Public Opinion Survey on the Acceptability of Crowdsourcing Basic Life Support for Out-of-Hospital Cardiac Arrest With the PulsePoint Mobile Phone App
Source: JMIR Mhealth Uhealth. 2017 May 17;5(5):e63. doi: 10.2196/mhealth.6926 (PMC5451638; doi:10.2196/mhealth.6926)
Supplement: Multimedia Appendix 2 [file mhealth_v5i5e63_app2.pdf]

TABLE 3: Weighted regression analysis for USA population

PREDICTOR VARIABLES		COMFORT IN A PUBLIC SETTING Multivariable Model[a]		COMFORT IN A PRIVATE SETTING Multivariable Model[b]	
		OR (95%CI)	*P value*	*OR (95%CI)*	*P value*
**SEX**	Base: Female (vs. Male)	1.3 (0.81;2.2)	.26	1.7 (1.2;2.4)	.004
**EDUCATION LEVEL**	Base: High School or less (vs. Post secondary education)	1.0 (0.60;1.7)	.99	0.91(0.61;1.4)	.65
**HOUSAEHOLD INCOME**	Base: <$49,000				
	$50,000-$99,999	0.88 (0.53;1.46)	.63	0.95 (0.64;1.4)	.81
	$100,000 or more	1.1 (0.56;2.2)	.32	0.64 (0.39;1.1)	.079
**EMPLOYMENT TYPE**	Base: Not currently employed				
	Student or Military	3.1 (0.59;16)	.18	1.3 (0.53;3.4)	.54
	Employed	0.67 (0.38;1.2)	.17	0.85 (0.58;1.2)	.40
**CHILDREN**	Base: No children (vs. at least one child)	0.87 (0.48;1.5)	.63	1.3 (0.85;2.1)	.20
**AGE GROUP**	(Base: Age 18-34)				
	Age 35-54	1.5 (0.86;2.7)	.15	1.2 (0.77;1.9)	.43
	Age 55+	1.2 (0.61;2.5)	.57	0.84 (0.5;1.4)	.50
**MARITAL STATUS**	Base: Single, never married				
	Married or Co-habitating	1.5 (0.82;2.8)	.18	1.7 (1.1;2.8)	0.027
	Widowed, Separated, Divorced	1.0 (0.47;2.1)	1.0	1.6 (0.89;3.0)	0.12
**RACE**	Base: Caucasian (vs. Other)	1.3 (0.70;2.2)	.44	0.82(0.54;1.3)	0.37

a: Multivariable Model; Comfort in Public Setting: Omnibus F-test: F(12,1283)=1.28; p=0.22. Goodness of Fit F-test: F(9,1286)=0.77; p=0.64

b: **Multivariable Model**; Comfort in Private Setting: F(12;1283=2.0; p=0.02. Goodness of Fit F-test F(9,1286) =0.93; p=0.50
