# Supplementary material for: North American Public Opinion Survey on the Acceptability of Crowdsourcing Basic Life Support for Out-of-Hospital Cardiac Arrest With the PulsePoint Mobile Phone App
Source: JMIR Mhealth Uhealth. 2017 May 17;5(5):e63. doi: 10.2196/mhealth.6926 (PMC5451638; doi:10.2196/mhealth.6926)
Supplement: Multimedia Appendix 3 [file mhealth_v5i5e63_app3.pdf]

TABLE 4: Weighted regression analysis for Canadian population

	PREDICTOR VARIABLES	COMFORT IN A PUBLIC SETTING Multivariable Model[a]		COMFORT IN A PRIVATE SETTING Multivariable Model[b]	
		OR (95%CI)	*P value*	*OR (95%CI)*	*P value*
**SEX**	Base: Female (vs. Male)	1.2 (0.62;2.3)	.62	2.3 (1.3;3.9)	.003
**EDUCATION LEVEL**	Base: High School or less (vs. Post secondary education)	0.53 (0.25;1.1)	.10	0.79 (0.45;1.4)	.42
**HOUSAEHOLD INCOME**	Base: <$49,000				
	$50,000-$99,999	1.0 (0.52;2.1)	.92	0.51 (0.28;0.92)	.024
	$100,000 or more	2.3 (0.80;6.6)	.12	1.0 (0.47;2.1)	1.0
**EMPLOYMENT TYPE**	Base: Not currently employed				
	Student or Military	0.54 (0.11;2.5)	.43	0.91 (0.23;3.5)	.89
	Employed	1.0 (0.51;2.1)	.90	1.0 (0.43;2.3)	.99
**CHILDREN**	Base: No children (vs. at least one child)	1.4 (0.59;3.1)	.46	0.70 (0.41;1.2)	.21
**AGE GROUP**	(Base: Age 18-34)				
	Age 35-54	0.31 (0.10;0.90)	.032	1.4 (0.78;2.6)	.26
	Age 55+	0.43 (0.12;1.5)	.18	1.2 (0.46;2.9)	.30
**PRIMARY LANGUAGE SPOKEN**	Base: French (vs. English)	0.99 (0.46;2.1)	.97	0.88 (0.43; 1.8)	.72

a: Multivariable Model; Comfort in Public Setting: Omnibus F-test: F(10,942)=1.30; p=0.22. Goodness of Fit F-test: F(9,943)= 3097; p=<0.0001
b: **Multivariable Model**; Comfort in Private Setting: Omnibus F-test: F(10,942) =2.98; p=0.0011. Goodness of Fit F-test F(9,943) =1.6; p=0.12
